# Knowledge and attitudes toward advanced cardiac life support among healthcare professionals in Addis Ababa, Ethiopia: a multicenter cross-sectional study

**DOI:** 10.1186/s12245-026-01385-w

**Published:** 2026-09-18

**Authors:** Hiwot Shewangizaw, Mikiyas G. Teferi, Biruk T. Mengistie, Chernet T. Mengistie, Nardos Belayneh, Yoseph Teshome, Biruk Girma, Yohannes Feleke, Estrella Roffe, Getaw W. Hassen

**Affiliations:** 1 Department of Emergency Medicine and Critical Care, School of Medicine, College of Health Sciences, Addis Ababa University, Addis Ababa, Ethiopia; 2 School of Medicine, College of Health Sciences, Addis Ababa University, Addis Ababa, Ethiopia; 3 Department of Obstetrics and Gynecology, School of Medicine, College of Health Sciences, Addis Ababa University, Addis Ababa, Ethiopia; 4 Department of Emergency Medicine, New York Medical College, Metropolitan Hospital Center, New York, USA

**Keywords:** Advanced cardiac life support, Knowledge, Attitude, Healthcare professionals, Ethiopia

## Abstract

**Background:**

Advanced Cardiac Life Support (ACLS) supports the management of cardiac arrest and other life-threatening cardiovascular emergencies. Studies from resource-limited settings have identified persistent gaps in ACLS knowledge and attitudes among healthcare professionals.

**Methods:**

This multicenter institution-based cross-sectional study was conducted from June to November 2024; data were collected from October to November 2024. Four referral hospitals were selected by convenience, and eligible healthcare professionals within those hospitals were selected by computer-generated simple random sampling. Data were collected using a structured, self-administered questionnaire. Good knowledge was defined as at least 7 correct answers among 14 questions. Attitude was measured with 12 five-point Likert items; participants with an aggregate score at or above the sample mean were classified as having a positive attitude. Data were analyzed using SPSS version 28. Multivariable logistic regression identified factors associated with knowledge and attitude, with adjusted odds ratios (AORs) and 95% confidence intervals (CIs) reported.

**Results:**

Of 397 sampled healthcare professionals, 379 completed the survey (response rate, 95.5%). Overall, 178 (47.0%) had good ACLS knowledge and 164 (43.3%) had a positive attitude. Nurses had higher adjusted odds of good knowledge than physicians (AOR, 2.09; 95% CI, 1.26–3.42). Participants with MSc-level education had lower adjusted odds of good knowledge than degree holders (AOR, 0.38; 95% CI, 0.16–0.92). For positive attitude, lower adjusted odds were observed among participants with 0–23 months (AOR, 0.20; 95% CI, 0.049–0.84) or 60–120 months (AOR, 0.17; 95% CI, 0.04–0.71) of experience than among those with more than 120 months, and among diploma holders than among degree holders (AOR, 0.41; 95% CI, 0.21–0.78). Previous ACLS training was not significantly associated with either outcome.

**Conclusion:**

Fewer than half of participants had good ACLS knowledge or a positive attitude. These findings identify educational gaps and support consideration of accessible, competency-oriented ACLS education, accompanied by local evaluation of practical performance and patient outcomes.

**Trial registration:**

N/A

**Clinical trial number:**

Not applicable.

## Background

Cardiac arrest is a major cause of death worldwide, although precise global incidence estimates remain uncertain [1]. In the United States, approximately 600,000–700,000 cardiac arrests occur annually, including about 290,000 in-hospital cardiac arrests [2, 3]. Survival remains limited: fewer than one-quarter of patients with in-hospital cardiac arrest survive to hospital discharge, and survival after out-of-hospital cardiac arrest is generally below 10% [4, 5]. Evidence from African health systems also documents limited critical care resources, which can constrain responses to life-threatening emergencies [6]. These disparities increase the importance of timely, coordinated resuscitation.

Advanced Cardiac Life Support (ACLS) comprises coordinated interventions for cardiac arrest and other life-threatening cardiovascular emergencies, including early recognition, high-quality cardiopulmonary resuscitation (CPR), defibrillation when indicated, airway management, vascular access, and medication administration [7]. The questionnaire for this 2024 study was developed using the 2020 American Heart Association (AHA) guidelines and the 2023 focused update, which were the applicable AHA guidance during data collection [7–9]. The AHA issued updated Adult Advanced Life Support recommendations in 2025, after data collection was completed [10]. Prompt recognition and high-quality CPR remain central to survival, and delays in resuscitation are associated with worse outcomes [11, 12].

Knowledge of ACLS and related resuscitation principles remains uneven across settings. In Sri Lanka, 43.1% of surveyed medical students and medical officers were classified as having good advanced life support knowledge, while a North Kerala study found that 48.6% of healthcare professionals scored above 50% on a combined BLS and ACLS assessment [13, 14]. A Saudi Arabian study also identified gaps in technical CPR knowledge among health professions students [15]. In Ethiopia, a 2022 study at Felege Hiwot Referral Hospital reported poor ACLS knowledge among 59.5% of healthcare professionals and a negative attitude among 44.75% [16]. Low theoretical knowledge scores have also been reported among Ecuadorian nursing professionals [17]. Differences in study populations, instruments, and thresholds limit direct comparison, but the findings consistently identify areas for resuscitation education.

Recent multicenter evidence on ACLS knowledge and attitudes among healthcare professionals in Ethiopia remains limited. Earlier Ethiopian studies were predominantly conducted at single institutions, limiting their applicability across hospitals and clinical settings. This study therefore assessed ACLS knowledge, attitudes, and associated factors among healthcare professionals in four selected referral hospitals in Addis Ababa. The findings were intended to identify educational needs and inform future evaluation of ACLS training and clinical competency.

## Methods and materials

### Study design, setting, and period

An institution-based, multicenter cross-sectional study was conducted from June to November 2024. Protocol development, ethical review, and preparation of the data-collection tool occurred from June to September; data were collected from October to November 2024. The study included Tikur Anbessa Specialized Hospital (approximately 1,729 healthcare professionals), Zewditu Memorial Hospital (approximately 1,176), St. Paul’s Hospital Millennium Medical College (approximately 1,200), and ALERT Comprehensive Specialized Hospital (approximately 1,950). The hospitals were selected by convenience based on clinical workforce, tertiary emergency care capacity, accessibility, and feasibility. Together, they employed an estimated 6,055 healthcare professionals. Because the hospitals were not randomly selected, the sample was not a probability sample of all healthcare professionals in Addis Ababa.

### Study population and sampling

The target population comprised licensed healthcare professionals working in referral hospitals in Addis Ababa. The source population comprised professionals employed at the four selected hospitals, and the study population included professionals directly involved in patient care: physicians, nurses, anesthetists, and midwives.

A two-stage sampling approach was used. First, the four hospitals were selected by convenience. Second, eligible healthcare professionals within each hospital were selected by simple random sampling. Human resource departments supplied lists of eligible professionals; each person was assigned a unique identification number, and computer-generated random numbers were used until the allocated sample for each hospital was reached.

### Eligibility criteria

Inclusion criteria covered all licensed healthcare workers engaged in direct clinical practice within theselected hospitals.

Exclusion criteria were volunteers, staff on annual or sick leave during the data-collection period, and non-clinical professionals such as pharmacists, laboratory technologists, and radiology personnel.

### Sample size determination

The sample size was calculated with the single-population proportion formula, n = Z²p(1 − p)/d², using a 95% confidence level (Z = 1.96) and a 5% margin of error. A prevalence of good ACLS knowledge of 40.5% from a previous Ethiopian study produced *n* = 370. For attitude, the prior positive-attitude prevalence of 56.25% produced *n* = 378.16, rounded to 379; the larger estimate was retained [16]. Finite-population correction for the source population of 6,055 gave 379/(1 + 379/6,055) = 356.67, equivalent to 357 after rounding. The 10% nonresponse adjustment was applied to the unrounded corrected value: 356.67/0.90 = 396.3, which was rounded up to a target of 397. Of those sampled, 379 completed the survey, for a response rate of 95.5%.

### Data collection instruments and procedures

Data were collected using a pretested, structured, self-administered questionnaire in English and deployed electronically through KoboToolbox. The instrument was adapted from published ACLS knowledge and attitude studies and from the AHA guidance available during tool development [7–9, 16].

Questionnaire components:

1. Sociodemographic and professional variables: age, sex, marital status, profession, educational level, years of experience, current department, and previous ACLS training.
2. Knowledge assessment: 14 multiple-choice questions covering ACLS definitions, CPR technique, defibrillation parameters, shockable and nonshockable rhythms, medication dosing (epinephrine, amiodarone, and magnesium sulfate), reversible causes of cardiac arrest, and post-cardiac-arrest care.
3. Attitude assessment: 12 items rated on a five-point Likert scale (1 = strongly disagree to 5 = strongly agree), addressing motivation to learn, confidence in performing ACLS, teamwork, communication, and ethical awareness.

The questionnaire was adapted from published ACLS knowledge and attitude studies and was reviewed by two emergency medicine specialists for content relevance and clarity. It was pilot tested with 5% of eligible participants at a hospital that did not participate in the main study; minor wording changes were made after the pilot. Internal consistency of the attitude scale was acceptable (Cronbach’s α > 0.70). Because the knowledge questions assessed distinct factual domains, Cronbach’s alpha was not used as the primary reliability measure for the knowledge scale.

### Operational definitions

- Good knowledge: at least 7 correct answers among the 14 knowledge questions.
- Poor knowledge: fewer than 7 correct answers among the 14 knowledge questions.
- Positive attitude: an aggregate score at or above the sample mean on the 12 attitude items.
- Negative attitude: an aggregate score below the sample mean on the 12 attitude items.

Attitude responses were coded from 1 to 5 so that higher values represented a more favorable attitude. The 12 responses were summed for each participant, producing a possible score from 12 to 60. The sample mean of the aggregate score was used as the analytical cutoff. This data-derived threshold has not been externally validated and should not be interpreted as a clinical standard.

The dependent variables were knowledge status (good or poor) and attitude (positive or negative). Independent variables were age, sex, marital status, profession, educational level, total work experience, department, and previous ACLS training.

### Statistical analysis

Cleaned data were exported from KoboToolbox to Microsoft Excel and analyzed with IBM SPSS Statistics version 28. Categorical variables were summarized as frequencies and percentages, and continuous variables as means with standard deviations (SDs).

Associations between each independent variable and the outcomes were first examined with bivariable logistic regression. Variables with *p* < 0.25 were entered into multivariable logistic regression models to adjust for potential confounding. Results are reported as crude odds ratios (CORs) and adjusted odds ratios (AORs) with 95% confidence intervals (CIs). Model fit was assessed with the Hosmer–Lemeshow goodness-of-fit test, for which *p* > 0.05 indicated acceptable fit. Multicollinearity was assessed with the variance inflation factor (VIF), using VIF < 5 as the prespecified threshold.

### Ethical considerations

Ethical approval was obtained from the Emergency and Critical Care Medicine Ethics Review Committee of Addis Ababa University (Reference No: AAU/ECCM-031/2024), followed by authorization from the Addis Ababa Health Bureau. Written informed consent was obtained from all participants after an explanation of the study purpose and procedures. Participation was voluntary, and respondents could withdraw at any stage without penalty. The survey collected no personal identifiers; data were stored in password-protected files and used solely for research purposes.

## Results

### Participant response and characteristics

Of 397 sampled healthcare professionals, 379 completed the survey (response rate, 95.5%). The mean age was 29.18 ± 3.47 years, and 282 participants (74.4%) were aged 18–30 years. There were 219 male participants (57.8%), and 227 participants (59.9%) were single. The sample included 260 nurses (68.6%), 106 physicians (28.0%), 6 anesthetists (1.6%), and 7 midwives (1.8%). Regarding education, 289 participants (76.3%) held a bachelor’s degree, 51 (13.5%) a diploma, and 39 (10.3%) an MSc qualification. Work experience ranged from less than 2 years for 127 participants (33.5%) to more than 10 years for 11 (2.9%). Participants worked in the emergency department (227, 59.9%), ward (85, 22.4%), intensive care unit (54, 14.2%), or operating room (OR; 13, 3.4%). Among emergency department staff, 69.9% had 3 years or less of emergency experience. Overall, 137 participants (36.1%) reported previous ACLS training and 242 (63.9%) did not; 60.3% of emergency department staff reported no previous ACLS training (Table 1). The anesthetist, midwife, and more-than-10-years-experience subgroups were small, which reduced the precision of some regression estimates.

**Table 1:** Sociodemographic characteristics of study participants

Variables		Frequency (%)
Age	18–30	282(74.4)
	31–40	92(24.3)
	≥ 41	5 (1.3)
Sex	Male	219(57.8)
	Female	160(42.2)
Marital status	Single	227(59.9)
	Married	150(39.6)
	Widowed	2(0.5)
Profession	Physicians	106(28)
	Nurses	260(68.6)
	Anesthetists	6(1.6)
	Midwives	7(1.8)
Educational level	MSc	39(10.3)
	Degree	289 (76.3)
	Diploma	51(13.5)
Experience in months	0–23	127(33.5)
	24–59	149(39.3)
	60–120	92(24.3)
	Above 120	11(2.9)
Previous ACLS training	Yes	137(36.1)
	No	242(63.9)
Participants’ current working department	Emergency	227 (59.9)
	ICU	54 (14.2)
	Operating room (OR)	13(3.4)
	Ward	85(22.4)

Using the prespecified cutoff of at least 7 correct answers, 178 participants (47.0%) had good ACLS knowledge and 201 (53.0%) had poor knowledge. The mean score was 7.3 ± 2.2 of 14 (52.1%). The highest correct-response proportions were for the immediate intervention when a patient has no pulse (280, 73.9%), adult chest-compression depth (254, 67.0%), the amiodarone dose (236, 62.3%), and the epinephrine dose and frequency (196, 51.7%). The lowest proportions were for reversible causes of cardiac arrest (60, 15.8%) and the adult CPR compression rate (76, 20.1%). In addition, 106 participants (28.0%) correctly identified the ACLS abbreviation and 117 (30.9%) identified the maximum biphasic defibrillation energy (Table 2).

**Table 2:** Knowledge of healthcare professionals regarding ACLS

Questions	Knowledge level (with correctanswer and %)
Abbreviation of advanced cardiac life support	106(28)
What is your immediate intervention (treatment) if the victim hasno pulse?	280(73.9)
What is location of chest compression?	151(39.8)
What is depth of chest compression for adults?	254(67)
What is adult advanced cardiac life support maximum joule forshockable rhythm	117(30.9)
What are non-shockable rhythms?	160(42.2)
What are shockable rhythms?	136(35.9)
What is the CPR rate of compression for adults?	76 (20.1)
What are the reversible causes of cardiac arrest?	60(15.8)
What is the dose and frequency of adrenalin for advanced cardiac life support?	196(51.7)
What is the dose of amiodarone for advanced cardiac lifesupport?	236(62.3)
Is magnesium sulfate used for advanced cardiac life support?	83(21.9)
Which is included in post-cardiac arrest care?	162(42.7)

### Attitude assessment toward ACLS

**Fig. 1:**
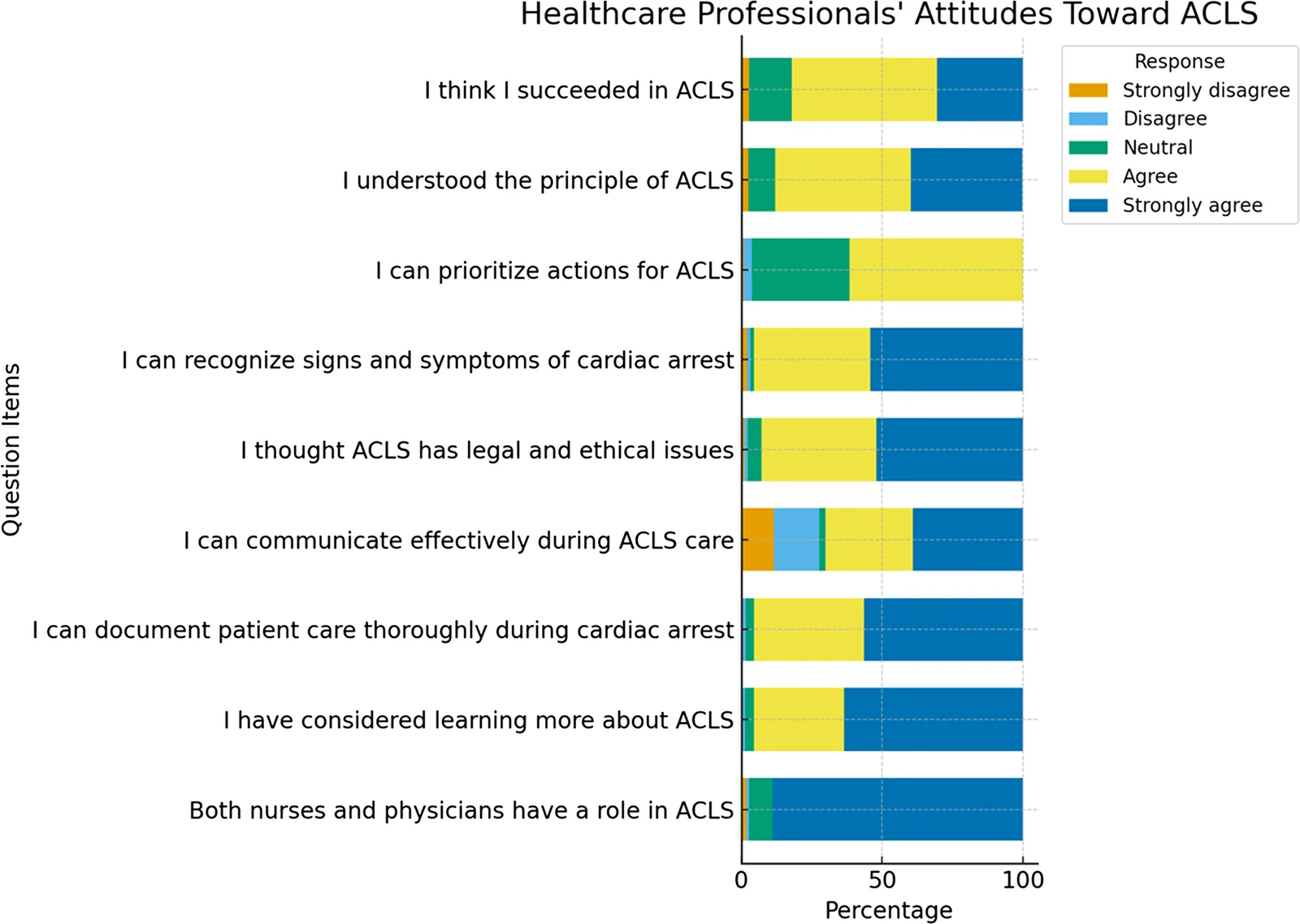
Distribution of responses to selected attitude statements about advanced cardiac life support. The figure presents agreement, neutrality, and disagreement across domains that include the perceived importance of ACLS, willingness to learn, communication, prioritization, confidence, and professional role recognition

Using the sample-mean cutoff described in the Methods, 164 participants (43.3%) were classified as having a positive attitude toward ACLS and 215 (56.7%) as having a negative attitude. Many participants agreed with statements about the importance of ACLS, willingness to learn, and professional roles in resuscitation. Responses were less favorable for confidence in communication and prioritizing actions during cardiac arrest. Classification was based on the aggregate score rather than on any single item (Fig. 1).

### Factors associated with knowledge toward ACLS

In the multivariable model, physicians, degree holders, and participants without previous ACLS training were the reference categories. Nurses had higher adjusted odds of good knowledge than physicians (AOR, 2.085; 95% CI, 1.26–3.42; *p* = 0.004). Participants with MSc-level education had lower adjusted odds than degree holders (AOR, 0.384; 95% CI, 0.159–0.92; *p* = 0.033). The MSc subgroup included 39 participants, and this estimate should therefore be interpreted cautiously. Previous ACLS training was not significantly associated with knowledge (AOR, 0.751; 95% CI, 0.49–1.15; *p* = 0.195). Adjusted associations for anesthetists and midwives were not statistically significant. Age, sex, department, and work experience were not significant predictors in the final model. The Hosmer–Lemeshow test indicated acceptable model fit (*p* > 0.05) (Table 3).

**Table 3:** Multivariable logistic regression for factors associated with good ACLS knowledge

Variable	Category	Good *n*	Poor *n*	AOR (95% CI)	*p*-value
**Profession**	Physician	38	68	Ref	—
	Nurse	134	126	2.085 (1.26–3.42)	0.004
	Anesthetist	1	5	0.411 (0.045–3.76)	0.430
	Midwife	5	2	3.941 (0.71–21.85)	0.110
**Educational level**	MSc	13	26	0.384(0.159–0.92)	0.033
	Degree	135	154	Ref	
	Diploma	30	21	0.806 (0.43–1.52)	0.506
Previous ACLS training	No	108	134	Ref	—
	Yes	70	67	0.751 (0.49–1.15)	0.195

Note: Wide confidence intervals reflect small subgroup sizes and should be interpreted with caution

### Factors associated with attitude toward ACLS

Profession, educational level, work experience, and previous ACLS training met the bivariable inclusion criterion of *p* < 0.25 and were entered into the multivariable model. Compared with participants who had more than 120 months of experience, those with 0–23 months had lower adjusted odds of a positive attitude (AOR, 0.20; 95% CI, 0.049–0.84; *p* = 0.028), as did those with 60–120 months (AOR, 0.168; 95% CI, 0.04–0.711; *p* = 0.015). The association for 24–59 months was not statistically significant. Diploma holders had lower adjusted odds of a positive attitude than degree holders (AOR, 0.407; 95% CI, 0.21–0.78; *p* = 0.007). Profession and previous ACLS training were not significantly associated with attitude. The reference group with more than 120 months of experience included only 11 participants, so the experience estimates require caution. The Hosmer–Lemeshow test indicated acceptable fit (*p* > 0.05), and VIF values were below 2.0 (Table 4).

**Table 4:** Multivariable logistic regression analysis of factors associated with positive attitude toward ACLS

Variable	Category	Positive	Negative	AOR (95% CI)	*p*-value
**Work experience (months)**	> 120	8	3	Ref	—
	0–23	53	74	0.20 (0.049–0.84)	0.028
	24–59	72	77	0.352 (0.086–1.436)	0.146
	60–120	31	61	0.168 (0.04–0.711)	0.015
**Educational level**	Degree	112	177	Ref	—
	MSc	20	19	0.549 (0.23–1.3)	0.17
	Diploma	32	19	0.407 (0.21–0.78)	0.007
**Profession**	Physician	35	71	Ref	—
	Nurses	123	137	1.90 (0.32–11.02)	0.47
	Anesthetist	4	2	2.84 (0.51–15.7)	0.231
	Midwives	2	5	8.04 (0.690–93.7)	0.096
Previous ACLS training	No	111	131	Ref	—
	Yes	53	84	0.679 (0.431–1.069)	0.095

Note: Wide confidence intervals reflect small subgroup sizes and should be interpreted cautiously

## Discussion

In this multicenter study of 379 healthcare professionals from four referral hospitals in Addis Ababa, 47.0% had good ACLS knowledge and 43.3% were classified as having a positive attitude. These proportions indicate important knowledge and attitude gaps in the selected institutions. Comparable studies have reported varied results because they used different populations, instruments, and classification thresholds [13, 14, 16, 17].

Knowledge varied substantially across resuscitation domains. Most participants identified the immediate response to pulselessness and the recommended chest-compression depth, but few identified the recommended compression rate or reversible causes of cardiac arrest. Compression rate and depth are clinically relevant components of high-quality CPR, although this questionnaire measured written knowledge rather than performance during resuscitation [18, 19].

The proportion classified as having a positive attitude was lower than the 56.25% reported in the Felege Hiwot study [16]. Many respondents endorsed individual statements about learning and professional responsibility even though fewer than half scored at or above the sample mean on the aggregate scale. This difference reflects the use of a relative, aggregate classification rather than a contradiction between individual responses and the overall result. Attitudes toward resuscitation vary across settings and instruments [20–22]. Because the sample-mean cutoff is data dependent and has not been externally validated, it may not capture all dimensions of confidence, motivation, or perceived role.

Nurses had higher adjusted odds of good ACLS knowledge than physicians. This differs from the Felege Hiwot study, in which physicians had higher knowledge scores [16]. Unequal professional-group sizes and differences in departmental distribution may have influenced the association; nurses constituted most of the present sample and many worked in the emergency department. The lower adjusted odds observed among participants with MSc-level education were also unexpected. Only 39 participants were in this subgroup, and the confidence interval was relatively wide. The result may reflect the small subgroup, residual confounding, or sampling variation and should not be interpreted as evidence that postgraduate education reduces ACLS knowledge. An Ethiopian systematic review of basic life support competency generally found higher educational attainment to be associated with better knowledge, further supporting cautious interpretation of the present finding [23].

Previous ACLS training was not significantly associated with knowledge or attitude in the adjusted models. This finding does not show that training is ineffective. The study was cross-sectional and recorded training as a binary exposure; it did not assess time since training, course type or duration, certification status, refresher training, simulation exposure, number of previous courses, or training quality. These differences could have obscured an association. A systematic review found that advanced life support knowledge and skills commonly decline after training, with skills often declining sooner than knowledge [24]. The present data cannot distinguish training decay from differences in course content, participant characteristics, or other unmeasured factors.

Nearly two-thirds of participants reported no previous ACLS training. This finding identifies limited training exposure within the selected hospitals but does not establish the effectiveness of any particular educational intervention. Evidence from randomized studies suggests that simulation-based methods, refresher activities, and changes in course delivery can support retention of advanced resuscitation skills, although the evidence is heterogeneous and does not identify a single optimal approach [25].

Hospitals may consider expanding access to structured ACLS education and periodic competency assessment, with equitable opportunities across professional groups and experience levels. Any program should be evaluated locally with objective measures of practical performance. Evidence outside this study includes observational associations between advanced life support course participation and selected resuscitation outcomes, but it does not establish that expanding training in these hospitals would improve patient outcomes [26]. Current resuscitation education guidance supports deliberate practice, feedback, and approaches designed to maintain competency over time [27]. Prospective studies should assess training delivery, skill retention, clinical performance, and patient outcomes in resource-limited settings.

This study has several limitations. Its cross-sectional design precludes causal inference, and self-reported information may be affected by recall or social desirability bias. Written knowledge questions do not measure practical ACLS skill or team performance. The four hospitals were selected by convenience and may differ from other Addis Ababa hospitals in staffing, training opportunities, case mix, resources, and institutional policies. The findings therefore should not be generalized to all healthcare professionals in Addis Ababa or to rural and primary care settings. Small numbers of anesthetists, midwives, MSc-level participants, and professionals with more than 10 years of experience reduced the precision and stability of some regression estimates. The sample-mean attitude cutoff was data derived and not externally validated. Training was measured only as a yes-or-no variable, without information on timing, content, duration, certification, refreshers, simulation, course frequency, or quality. These limitations should be considered when interpreting the observed associations.

## Conclusion

Fewer than half of the participating healthcare professionals had good ACLS knowledge or were classified as having a positive attitude. Nurses had higher adjusted odds of good knowledge than physicians, while the lower adjusted odds observed in the small MSc subgroup were unexpected and require cautious interpretation. Educational level and selected work-experience categories were associated with attitude. Because the study used a written questionnaire and a cross-sectional design, it cannot determine practical ACLS competence, the effectiveness of previous training, or effects on patient outcomes. Hospitals may consider regular, competency-oriented ACLS education, accompanied by objective evaluation of skill retention, clinical performance, and patient outcomes.

## Data Availability

The datasets generated and/or analyzed during the current study are available from the corresponding author upon reasonable request.

## Abbreviations

ACLS: Advanced cardiac life support
AHA: American heart association
AOR: Adjusted odds ratio
BLS: Basic life support
CI: Confidence interval
CPR: Cardiopulmonary resuscitation
COR: Crude odds ratio
ICU: Intensive care unit
IHCA: In-hospital cardiac arrest
LMICs: Low- and middle-income countries
MSc: Master of science
OHCA: Out-of-hospital cardiac arrest
OR: Operating room
SD: Standard deviation
SPSS: Statistical package for the social sciences
USA: United states of america
VIF: Variance inflation factor
